# Functional Outcomes of Endoscopic Sinus Surgery: A Prospective Cohort Study Using the Sino-Nasal Outcome Test-16 Questionnaire

**DOI:** 10.7759/cureus.69322

**Published:** 2024-09-13

**Authors:** Khalid A Shehri, Salam Sait, Sarah Alamoudi, Yahya Khubrani, Abdullah Bahakim

**Affiliations:** 1 Otolaryngology Head and Neck Surgery, King Abdulaziz University Hospital, Jeddah, SAU; 2 Otolaryngology Head and Neck Surgery, King Abdullah Medical City, Makkah, SAU; 3 Otolaryngology Head and Neck Surgery, King Faisal Specialist Hospital and Research Center, Jeddah, SAU

**Keywords:** chronic rhinosinusitis, endoscopic sinus surgery, nasal polyps, quality of life, questionnaire, sinonasal outcome test

## Abstract

Background: Chronic rhinosinusitis affects a large portion of the adult population, and its symptoms can be burdensome to patients’ quality of life. Functional endoscopic sinus surgery (FESS) is usually required after medical therapies fail.

Objective: To analyze the outcomes of FESS in a tertiary hospital using the modified Arabic Sinonasal Outcome Test.

Methods: This prospective cohort study involved administering an electronic questionnaire, the modified Arabic Sinonasal Outcome Test-16, pre-operatively to patients diagnosed with chronic rhinosinusitis who underwent FESS. This test was subsequently resent six weeks post-operatively. Scores were calculated by adding the total score of the 16 questions. The lowest possible score was 0, and the highest score was 48. Pre- and post-operative scores were compared.

Results: Twenty-eight patients were included, with a mean age of 37.3±14.6. Our findings showed that FESS significantly improved all symptoms except cough and exophthalmos (p < 0.001). Nose congestion had the highest improvement score, with 78.6% of patients experiencing better symptoms. Lack of good night sleep was the second most ameliorated symptom, with 75% of patients showing improvement.

Conclusion: Modified Arabic Sinonasal Outcome Test scores were significantly improved after FESS. Therefore, primary treatment with FESS should be considered for patients with chronic rhinosinusitis with nasal polyposis. However, further studies are recommended to investigate the long-term benefits of FESS and the impact of comorbidities, such as asthma and allergic rhinitis, on outcomes.

## Introduction

Chronic rhinosinusitis (CRS) is one of the most common health conditions affecting the adult population, with a prevalence of 5%-12%. Improving patient care in CRS remains a priority for healthcare providers. Patients mainly present with nasal obstruction, nasal discharge, anosmia or hyposmia, facial pain or pressure, and/or sleep disturbance [1-3]. The primary aim in the management of CRS is symptomatic control. To date, patient-reported outcome measurements (PROMs) such as the Rhinosinusitis Outcome Measure-31, Rhinosinusitis Quality of Life Survey, and Sino-Nasal Outcome Test-20 (SNOT-20), which were later modified into SNOT-22 and SNOT-16, are considered valid and reliable tools for evaluating patient-reported symptoms, the effects of CRS on sleep, and its phycological impact [4-6]. Research using PROMs to evaluate quality of life (QoL) post-sinus surgery reported varying outcomes, with most reporting significant improvement [2,3,7-10].

Despite the fact that medical treatment remains the mainstay of CRS management, functional endoscopic sinus surgery (FESS) is usually required in refractory cases to achieve better outcomes [3]. This study aimed to analyze FESS outcomes in a tertiary hospital, using the modified Arabic Sino-Nasal Outcome Test (MA-SNOT).

## Materials and methods

Study setting and participants

This prospective cohort study was conducted at King Abdulaziz University Hospital (KAUH) a tertiary hospital in Jeddah, Saudi Arabia, from April to December 2022. An electronic questionnaire was sent to all the patients who were diagnosed with chronic rhinosinusitis and planned for FESS; subsequently, the same questionnaire was resent to each patient six weeks after the surgery. Patients were excluded if they had not completed both pre-operative and post-operative SNOT-16 evaluations.

Questionnaire 

The electronic questionnaire was self-administered and comprised two parts. The first part included demographic data, such as sex, age, nationality, marital status, smoking status, prior FESS, and prior chronic conditions. The second part aimed to assess the QoL using the SNOT-16, which is a validated patient-reported outcome measure established to delineate the presence and severity of sinonasal disorders. The questionnaire included questions on CRS severity. Magnitude scores were based on a scale of 0 to 3, with 0 indicating no problems with the given symptom and 3 indicating maximal problems. Absolute scores were calculated by adding the total scores for each of the 16 questions. Thus, the lowest score was 0, and the highest was 48.

The SNOT was first described by Anderson et al. in 1999 [11] and has been widely used in many different populations worldwide for the quantitative description of sinonasal disorders. This approach has been shown to be effective in cross-cultural settings in other studies. This questionnaire was translated into Arabic and validated by Marglani et al. in 2011 [12]. The English version of the questionnaire used can be found in the Appendix (Table 4).

Statistical analysis 

Statistical analysis was conducted using the Statistical Package for the Social Sciences (IBM SPSS, version 29.0). The Shapiro-Wilk test was used to evaluate the distribution of continuous variables. Means and standard deviations were used to summarize continuous variables, whereas proportions were used to summarize categorical variables. Student’s t-test was used to compare the post-operative MA-SNOT between demographic groups. Dividing the subgroups by sex and marital status, a paired t-test was used to assess the difference between pre- and post-operative MA-SNOT scores after applying the assumptions. The pre- and post-operative components of the MA-SNOT were compared using the Wilcoxon test for paired ordinal variables. The level of significance was set at p < 0.05.

## Results

A total of 28 patients who underwent FESS were analyzed. The mean age was 37.3 ± 14.6. Of all patients, 16 (57.1%) were married. The male-to-female ratio was nearly balanced, comprising 13 (46.4%) males and 15 (53.6%) females. The study revealed that most of the patients had no smoking history, with 22 (78.6%) of them reporting no history of smoking. Additionally, a significant proportion of the patients had a prior surgical history; nine(32.1%) had undergone prior FESS, and three (10.7%) had undergone prior septoplasty. The demographic characteristics and medical histories are shown in Table 1. Computed tomography (CT) was performed pre-operatively and revealed polyps in 21 (75%) cases and a deviated nasal septum (DNS) in 15 (53.6%) cases.

**Table 1:** Demographic characteristics and past medical history of patients who underwent FESS FESS: functional endoscopic sinus surgery, DM: diabetes mellitus, HTN: hypertension.

Variable	No.	Percentage
Sex		
Male	13	46.4%
Female	15	53.6%
Nationality		
Saudi	27	96.4%
Non-Saudi	1	3.6%
Marital status		
Single	12	42.9%
Married	16	57.1%
Smoking status		
No	22	78.6%
Yes	3	10.7%
Ex-smoker	3	10.7%
Medical history		
DM or HTN	4	14.3%
Allergic rhinitis	16	57.1%
Asthma	14	50%
Anosmia secondary to neurological insult	5	17.9%
Headache	5	17.9%
Cataract or glaucoma	7	25%
Surgical history		
Prior septoplasty	3	10.7%
Prior FESS	9	32.1%

The mean pre- and post-operative MA-SNOT scores in regard to patients’ gender and marital status were compared. Both male and female patients and single and married patients showed significant improvement (p-value < 0.001). Furthermore, a statistically significant difference was observed in the pre- and post-operative MA-SNOT scores among smokers, non-smokers, and ex-smokers.

The impact of prior FESS on the mean pre-operative and post-operative MA-SNOT scores was analyzed. Whether patients had prior FESS or not, all exhibited statistically significant improvement in their scores after the surgery. For patients who had prior FESS, the mean pre-operative MA-SNOT score was 28.11 ± 10, while the mean post-operative score was 12.22 ± 12.06. For patients who did not have prior FESS, the mean pre-operative score was 28.37 ± 9.26, and the mean post-operative score was 15.21 ± 8.64. Our analysis found that post-operative MA-SNOT scores were significantly lower compared to pre-operative scores across all subgroups. 

**Figure 1:**
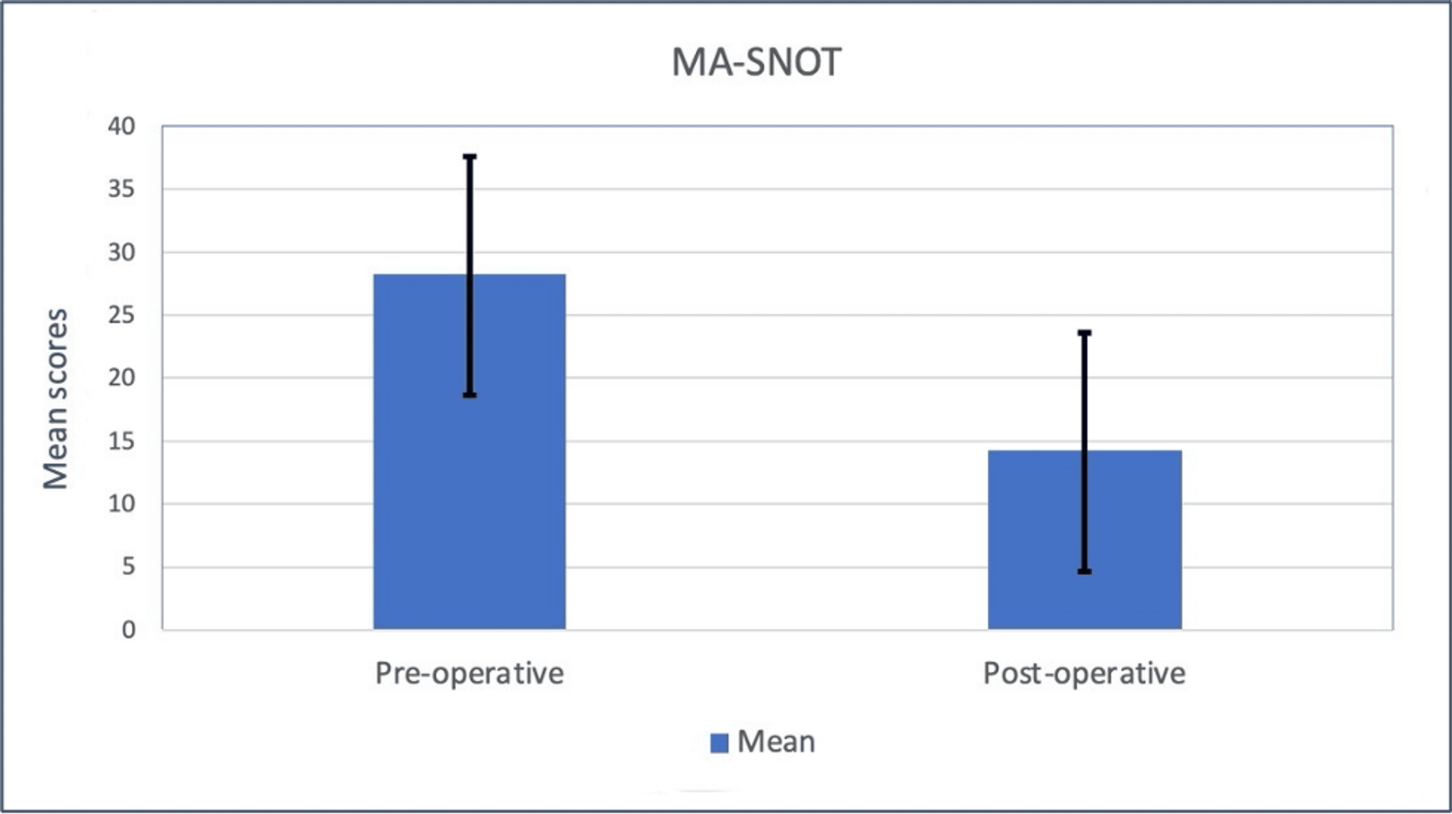
Comparison between pre-operative and post-operative MA-SNOT means among patients who underwent FESS MA-SNOT: modified Arabic Sinonasal Outcome Test, FESS: functional endoscopic sinus surgery.

Further statistical analysis revealed no significant differences when comparing post-operative MA-SNOT scores between any demographic group. A subgroup analysis of the MA-SNOT is shown in Table 2. In addition, this study investigated pre- and post-operative MA-SNOT scores in patients who underwent FESS. The pre- and post-operative mean scores were 28.7 ± 9.2 and 13.5 ± 9.0, respectively. The difference between the two scores was statistically significant (p < 0.001). The results are shown in Figure 1.

**Table 2:** Comparison between pre- and post-FESS among the demographic subgroups MA-SNOT: modified Arabic Sinonasal Outcome Test, FESS: functional endoscopic sinus surgery. p-value calculated using paired t-test; p < 0.05 was considered 'significant'.

	Pre-operative MA-SNOT	Post-operative MA-SNOT	p-value
	Mean±SD	Mean±SD	
Sex			
Male	26.62 ± 9.99	12.92 ± 8.97	<0.001
Female	29.73 ± 8.77	15.4 ± 10.53	<0.001
Marital status			
Single	31.83 ± 7.69	17.17 ± 10.01	<0.001
Married	25.63 ± 9.76	12.06 ± 9.24	<0.001
Smoking			
Yes	32 ± 1.73	13.67 ± 8.5	0.03
No	26.95 ± 9.94	14.14 ± 10.69	<0.001
Ex-smoker	34.33 ± 6.11	15.67 ± 2.31	0.01
Prior FESS			
Yes	28.11 ± 10	12.22 ± 12.06	0.002
No	28.37 ± 9.26	15.21 ± 8.64	<0.001

Our findings showed that the FESS significantly improved most symptoms. Statistically significant improvements were observed in all symptoms except cough and exophthalmos. The symptom that showed the greatest improvement was blockage/congestion of the nose, with 78.6% of patients experiencing improvement. Lack of good night sleep was the second most improved symptom, with 75% of the patients showing improvement (p-value < 0.001). Details of the percentage of patients who experienced improvement in each symptom, the mean score before and after surgery, and the p-values for each symptom are shown in Table 3.

**Table 3:** Comparison of MA-SNOT items pre- and post-FESS among the sample MA-SNOT: modified Arabic Sinonasal Outcome Test, FESS: functional endoscopic sinus surgery. p-value calculated using Wilcoxon test; p < 0.05 was considered 'significant'.

Item	Improved (%)	Pre-FESS Mean ± SD	Post-FESS Mean ± SD	p-value*
Need to blow nose	19 (67.9%)	2.5 ± 0.7	1.5 ± 0.9	<0.001
Sneezing	18 (64.3%)	1.7 ± 1	1.0 ± 0.9	0.003
Runny nose	21 (75%)	2.3 ± 0.9	1.1 ± 0.7	<0.001
Blockage/congestion of nose	22 (78.6%)	2.6 ± 0.7	1.0 ± 1	<0.001
Loss of sense of taste/smell	17 (60.7%)	2.1 ± 1.2	1.1 ± 1	0.002
Cough	9 (32.1%)	0.9 ± 1.1	0.6 ± 0.8	0.198
Itching of nose, throat, or eyes	18 (64.3%)	1.8 ± 0.9	0.9 ± 1	0.002
Post-nasal discharge	15 (53.6%)	2.1 ± 0.9	1.2 ± 0.9	0.003
Ear fullness	19 (67.9%)	1.7 ± 1	0.8 ± 0.9	0.001
Facial pain/pressure	16 (57.1%)	2.0 ± 1	1.2 ± 1.1	0.002
Lack of a good night’s sleep	21 (75%)	2.2 ± 0.9	0.6 ± 1	<0.001
Waking up tired	16 (57.1%)	1.9 ± 1	0.9 ± 0.9	<0.001
Fatigue	18 (64.3%)	2.1 ± 1	1.0 ± 1	<0.001
Double vision	11 (39.3%)	0.9 ± 1	0.4 ± 0.7	0.022
Weak vision	12 (42.9%)	1 ± 0.9	0.5 ± 0.9	0.014
Exophthalmos	9 (32.1%)	0.6 ± 0.9	0.4 ± 0.9	0.697

## Discussion

Functional endoscopic sinus surgery is a minimally invasive procedure used to treat sinonasal disorders [13]. This study encompassed 75% of polyp cases, and 53.6% were diagnosed with a DNS, as verified via CT scan. This study aimed to analyze the outcomes of FESS and improvement in symptom severity after surgical intervention. We used the MA-SNOT to collect data from participants. The results of our study indicate that FESS leads to a significant improvement in symptom severity. These findings were confirmed by a substantial reduction in MA-SNOT scores following surgery. Importantly, sinonasal disorders often significantly affect the QoL of patients and usually require surgical intervention [14]. Although FESS is a commonly used surgical option for most sinonasal disorders, surgical treatment often does not ensure a permanent cure for nasal polyposis [15-17]. Therefore, determining the potential surgical outcomes after surgery can better guide the future management of such patients. Currently, several patient-reported outcome measures are used, including the SNOT. This test was designed by Anderson et al. in 1999 and comprises multiple questions that help determine the QoL after the incidence of any nasal disease [11]. Initially, the SNOT, a 20-item validation tool, was enhanced by adding two items (nasal blockage, change in smell/taste), creating the SNOT-22. This version includes 22 items assessing rhinological, ear and facial symptoms, sleep function, and psychological impacts on nasal and paranasal diseases [4]. Its validity is established across various populations and languages, such as Greek, Portuguese, Czech, Hebrew, and English [4,18,19]. Morley and Sharp's study reviewed 15 sinonasal outcome systems, identifying the SNOT-22 as superior in validity, sensitivity, and reliability [20]. Additionally, it is user-friendly.

Alanazy et al. validated the Arabic version of SNOT-22 previously in their study [21]. Their findings showed that the Arabic version of the SNOT-22 could help differentiate between healthy volunteers and those with disease (p < 0.001). Additionally, the MA-SNOT has been used to evaluate the features of post-coronavirus disease 2019 parosmia as well. The present study utilized the MA-SNOT tool, which was initially validated by Marglani et al. [12], who showed that the mean score of this 16-item SNOT tool was significantly lower in healthy controls (2.6) than in the patient group (30.9) (p < 0.001). Unlike the present study, they did not investigate the pre- and post-operative MA-SNOT scores of the participants and had a very narrow scope of investigation. Here, we found a significant difference in the pre- and post-operative SNOT scores in patients (p < 0.001). Aligning with Adnane et al.'s findings, we observed significant SNOT score differences pre- and six months post-surgery (p < 0.001) and between treatment and control groups [22]. Utilizing the Moroccan Arabic 22-Item SNOT, we noted a decrease in pre-operative mean score from 28.7 ± 9.2 to 13.5 ± 9.0 post-operatively, echoing Asiri and Alokby's MA-SNOT findings [23]. Similarly, a Brazilian SNOT variant reported pre-operative, post-operative, and non-sinonasal disease scores of 62.39, 23.09, and 11.42, respectively (p < 0.0001) [24].

Hopkins et al. conducted a comprehensive study involving 3,128 adult patients who underwent sinonasal surgery across 87 hospitals within the National Health Service in England and Wales [4]. The primary objective of this study was to validate the SNOT tool, confirming a significant decrease (p < 0.001) in SNOT-22 scores three months after the surgical intervention [4]. These findings enhance the FESS knowledge base, affirming its efficacy in patient outcomes. Furthermore, our results substantiate MA-SNOT's utility in assessing patient-reported outcomes post-FESS [19,20,23].

Our findings demonstrated that the FESS significantly improved the majority of symptoms, with the highest improvement observed in the quality of sleep in 75% of the participants (p<0.001). Our findings align with those of Alt et al., who reported that FESS significantly improved all quality-of-life parameters in patients with chronic rhinosinusitis (p < 0.05) [25]. Similarly, Rotenberg and Pang reported that sleep outcomes, including sleep quality and duration, significantly improved after FESS (p < 0.01) [26]. However, they did not record a significant change in nasal obstruction scores [26]. Nonetheless, in the present study, we observed a significant improvement in nasal blockage (p < 0.001). Here, we did not observe any significant improvement in cough or exophthalmos after FESS. Our findings contradict those of Djukic et al., who reported significant improvement in cough (p < 0.001) six months post-surgery [27]. Regarding sleep-related outcomes after FESS, our results are supported by those of Uz et al., who investigated the pattern and quality of sleep in patients with nasal polyps [28]. This is important because almost 75% of our participants had nasal polyps. Finally, Uz et al. showed that the total apnea index and apnea-hypopnea index significantly improved after surgery (25.4 to 7.8 and 13.3 to 11.2, respectively) [28].

The current study employed the MA-SNOT-16, a modified SNOT-22, for evaluating postsurgical outcomes post-FESS. This previously validated tool improves objectivity and comparability in results. Nonetheless, limitations exist, including scarce comparative published data and potential bias from the self-reported symptom severity and improvement assessments.

## Conclusions

Functional endoscopic sinus surgery is a safe and effective treatment for patients with chronic rhinosinusitis with nasal polyposis. In this study, FESS was found to significantly improve post-operative MA-SNOT scores, with no significant differences between the demographic groups. This suggests that FESS should be considered a primary treatment option for patients with chronic rhinosinusitis with nasal polyposis. However, further studies should investigate the long-term benefits of FESS and the impact of comorbidities such as asthma and allergic rhinitis on FESS outcomes. Clinicians need to consider these findings when discussing treatment options with patients, as FESS can provide an effective solution for chronic rhinosinusitis with nasal polyposis.

## Appendices

On a scale of 0 (none) to 5 (very severe), please rate each of the items below based on how ‘bad’ it is when you experience it and how frequently it happens:

**Table 4:** Sino-Nasal Outcome Questionnaire-16 (English version)

Item	None	Very mild	Mild	Moderate	Severe	Very severe	Score
Need to blow nose	0	1	2	3	4	5	
Sneezing	0	1	2	3	4	5	
Runny nose	0	1	2	3	4	5	
Blockage/congestion of nose	0	1	2	3	4	5	
Loss of sense of taste/smell	0	1	2	3	4	5	
Cough	0	1	2	3	4	5	
Itching of nose, throat, or eyes	0	1	2	3	4	5	
Post-nasal discharge	0	1	2	3	4	5	
Ear fullness	0	1	2	3	4	5	
Facial pain/pressure	0	1	2	3	4	5	
Lack of a good night’s sleep	0	1	2	3	4	5	
Waking up tired	0	1	2	3	4	5	
Fatigue	0	1	2	3	4	5	
Double vision	0	1	2	3	4	5	
Weak vision	0	1	2	3	4	5	
Exophthalmos	0	1	2	3	4	5	
Total score							

## References

[1] Hastan D, Fokkens WJ, Bachert C (2011). Chronic rhinosinusitis in Europe: an underestimated disease. A GA²LEN study. Allergy.

[2] Kennedy JL, Hubbard MA, Huyett P, Patrie JT, Borish L, Payne SC (2013). Sino-nasal outcome test (SNOT-22): a predictor of postsurgical improvement in patients with chronic sinusitis. Ann Allergy Asthma Immunol.

[3] Steinke JW, Payne SC, Tessier ME, Borish LO, Han JK, Borish LC (2009). Pilot study of budesonide inhalant suspension irrigations for chronic eosinophilic sinusitis. J Allergy Clin Immunol.

[4] Hopkins C, Gillett S, Slack R, Lund VJ, Browne JP (2009). Psychometric validity of the 22-item Sinonasal Outcome Test. Clin Otolaryngol.

[5] Dietz de Loos DA, Segboer CL, Gevorgyan A, Fokkens WJ (2013). Disease-specific quality-of-life questionnaires in rhinitis and rhinosinusitis: review and evaluation. Curr Allergy Asthma Rep.

[6] Garbutt J, Spitznagel E, Piccirillo J (2011). Use of the modified SNOT-16 in primary care patients with clinically diagnosed acute rhinosinusitis. Arch Otolaryngol Head Neck Surg.

[7] Soler ZM, Jones R, Le P, Rudmik L, Mattos JL, Nguyen SA, Schlosser RJ (2018). Sino-Nasal outcome test-22 outcomes after sinus surgery: a systematic review and meta-analysis. Laryngoscope.

[8] Yancey KL, Lowery AS, Chandra RK, Chowdhury NI, Turner JH (2019). Advanced age adversely affects chronic rhinosinusitis surgical outcomes. Int Forum Allergy Rhinol.

[9] Hoseini SM, Saedi B, Aghazadeh K (2012). Meticulous endoscopic sinus surgery to prevent recurrence of massive nasal polyposis. J Laryngol Otol.

[10] Briggs RD, Wright ST, Cordes S, Calhoun KH (2004). Smoking in chronic rhinosinusitis: a predictor of poor long-term outcome after endoscopic sinus surgery. Laryngoscope.

[11] Anderson ER, Murphy MP, Weymuller EA Jr (1999). Student Research Award 1998: Clinimetric evaluation of the Sinonasal Outcome Test-16. Otolaryngol Head Neck Surg.

[12] Marglani O, Alherabi A, Fattah T (2011). The introduction and validation of a modified Arabic Sino-Nasal outcome test (MA-SNOT). Pan Arab J Rhinol.

[13] El-Sayed TR, Goda MFA, El-Gerby KM (2022). Multislice CT in sinonasal polypi; functional endoscopic sinus surgery correlation. Egypt J Hosp Med.

[14] Sedaghat AR, Kuan EC, Scadding GK (2022). Epidemiology of chronic rhinosinusitis: prevalence and risk factors. J Allergy Clin Immunol Pract.

[15] Stankiewicz JA, Lal D, Connor M, Welch K (2011). Complications in endoscopic sinus surgery for chronic rhinosinusitis: a 25-year experience. Laryngoscope.

[16] Adnane C, Adouly T, Zouak A, Mahtar M (2015). Quality of life outcomes after functional endoscopic sinus surgery for nasal polyposis. Am J Otolaryngol.

[17] Lachanas VA, Tsea M, Tsiouvaka S, Hajiioannou JK, Skoulakis CE, Bizakis JG (2014). The sino-nasal outcome test (SNOT)-22: validation for Greek patients. Eur Arch Otorhinolaryngol.

[18] Schalek P, Otruba L, Hahn A (2010). Quality of life in patients with chronic rhinosinusitis: a validation of the Czech version of SNOT-22 questionnaire. Eur Arch Otorhinolaryngol.

[19] Shapira Galitz Y, Halperin D, Bavnik Y, Warman M (2016). Sino-Nasal Outcome Test-22: translation, cross-cultural adaptation, and validation in Hebrew-Speaking Patients. Otolaryngol Head Neck Surg.

[20] Morley AD, Sharp HR (2006). A review of sinonasal outcome scoring systems: which is best?. Clin Otolaryngol.

[21] Alanazy F, Dousary SA, Albosaily A, Aldriweesh T, Alsaleh S, Aldrees T (2018). Psychometric Arabic Sino-Nasal Outcome Test-22: validation and translation in chronic rhinosinusitis patients. Ann Saudi Med.

[22] Adnane C, Adouly T, Oubahmane T (2016). Psychometric validation of a Moroccan version of the 22-Item Sino-Nasal Outcome Test. Otolaryngol Head Neck Surg.

[23] Asiri M, Alokby G (2019). Validation and cross-cultural adaptation of the Sinonasal Outcome Test (SNOT)-22 for the Arabian patient population. Cureus.

[24] Kosugi EM, Chen VG, Fonseca VM, Cursino MM, Mendes Neto JA, Gregório LC (2011). Translation, cross-cultural adaptation and validation of SinoNasal Outcome Test (SNOT): 22 to Brazilian Portuguese. Braz J Otorhinolaryngol.

[25] Alt JA, DeConde AS, Mace JC, Steele TO, Orlandi RR, Smith TL (2015). Quality of life in patients with chronic rhinosinusitis and sleep dysfunction undergoing endoscopic sinus surgery: a pilot investigation of comorbid obstructive sleep apnea. JAMA Otolaryngol Head Neck Surg.

[26] Rotenberg BW, Pang KP (2015). The impact of sinus surgery on sleep outcomes. Int Forum Allergy Rhinol.

[27] Djukic V, Dudvarski Z, Arsovic N, Dimitrijevic M, Janosevic L (2015). Clinical outcomes and quality of life in patients with nasal polyposis after functional endoscopic sinus surgery. Eur Arch Otorhinolaryngol.

[28] Uz U, Günhan K, Yılmaz H, Ünlü H (2017). The evaluation of pattern and quality of sleep in patients with chronic rhinosinusitis with nasal polyps. Auris Nasus Larynx.

